# Network Inference and Maximum Entropy Estimation on Information Diagrams

**DOI:** 10.1038/s41598-017-06208-w

**Published:** 2017-08-01

**Authors:** Elliot A. Martin, Jaroslav Hlinka, Alexander Meinke, Filip Děchtěrenko, Jaroslav Tintěra, Isaura Oliver, Jörn Davidsen

**Affiliations:** 1 Complexity Science Group, Department of Physics and Astronomy, University of Calgary, Calgary, Alberta T2N 1N4 Canada; 2 Institute of Computer Science, The Czech Academy of Sciences, Pod vodarenskou vezi 2, 18207 Prague, Czech Republic; 3 National Institute of Mental Health, Topolová, 748, 250 67 Klecany Czech Republic; 4 Institute of Psychology, The Czech Academy of Sciences, Prague, Czech Republic; 5 Institute for Clinical and Experimental Medicine, Videnska 1958/9, 140 21 Prague, Czech Republic

## Abstract

Maximum entropy estimation is of broad interest for inferring properties of systems across many disciplines. Using a recently introduced technique for estimating the maximum entropy of a set of random discrete variables when conditioning on bivariate mutual informations and univariate entropies, we show how this can be used to estimate the *direct* network connectivity between interacting units from observed activity. As a generic example, we consider phase oscillators and show that our approach is typically superior to simply using the mutual information. In addition, we propose a nonparametric formulation of connected informations, used to test the explanatory power of a network description in general. We give an illustrative example showing how this agrees with the existing parametric formulation, and demonstrate its applicability and advantages for resting-state human brain networks, for which we also discuss its direct effective connectivity. Finally, we generalize to continuous random variables and vastly expand the types of information-theoretic quantities one can condition on. This allows us to establish significant advantages of this approach over existing ones. Not only does our method perform favorably in the undersampled regime, where existing methods fail, but it also can be dramatically less computationally expensive as the cardinality of the variables increases.

## Introduction

Statistical mechanics is based on the assumption that the most probable state of a system is the one with maximal entropy. This was later shown by Jaynes^1^ to be a general property of statistical inference — the least biased estimate must have the maximum entropy possible given the constraints, otherwise one implicitly or explicitly assumes extra constraints. This has resulted in maximum entropy methods being applied widely outside of traditional statistical physics.

In particular as a recent development, much work has been devoted to applying maximum entropy methods to the study of complex systems from a network perspective. Inferring networks from dynamical time series has seen much attention^2–4^, with applications in such diverse fields as neuroscience^5–10^, genetics^11^, and the climate^12^, as well as for generic coupled oscillators^13^. While most methods are based on links representing some form of statistical dependence between the state variables of the individual subsystems, a wide range of alternative concepts have been also in use – see e.g. refs 14–19, and references therein. Maximum entropy methods have proved useful in this task of inferring interaction networks, e.g., between genes^20, 21^, species^22^, and economies of countries^23^. Additionally, they have proved useful in determining how well a system can be described by a network made from pairwise measurements in general^24–29^. However, these methods typically condition on cross-correlations, which are not capable of detecting nonlinear relationships or clearly identifying *direct* connections. In addition, the computational costs quickly become prohibitive as the number of discrete states the random variables can take on increases (i.e. the cardinality of the variables increases).

In order to overcome these difficulties we propose here a novel methodology that (i) explicitly takes into account nonlinear relationships, (ii) allows one to infer direct network connections, (iii) is much faster than other techniques for cardinalities greater than 2, and (iv) can be applied reliably even in the undersampled regime. It is based on the set-theoretic formulation of information theory and conditions on mutual informations^30^. Additionally, we use this methodology to construct a non-parametric estimate of connected informations, introduced in ref. 31. These are used to determine how well a system can be described by a network inferred from pairwise measurements, as well as to estimate the relevance of higher-order interactions in sets of variables. We use our technique to help resolve an outstanding issue of applying maximum entropy models to functional magnetic resonance imaging (fMRI) data, where past methods showed that pairwise measurements were a good representation of the data only when it was discretized to two states^32^. Here we show that discretizing to larger cardinalities does not appreciably affect results from our method, though it does for methods only conditioning on cross-correlations. We also show that our methodology gives robust and consistent estimates of the backbone of resting-state brain networks associated with the fMRI data.

The outline of our paper is as follows. In the section Method we introduce our method of entropy maximization and show how one can vastly increase the types of information-theoretic quantities that one can condition on using the method we introduced in ref. 30, as well as extend the method to continuous variables. Next, in the section Network Inference we show how to infer direct network connectivity using our method. Then in the section Estimating Connected Informations we show how our method can be used to estimate connected informations, and give a relevant example using fMRI data, for which we also investigate its effective network connectivity. Finally, in the section Computational Advantages we demonstrate various computational advantages of our technique. Relevant proofs are left to their own section at the end.

## Method

The bivariate mutual information can detect arbitrary interactions between two variables, and is only zero when the variables are pairwise independent^33^. In theory, conditioning on mutual informations can be accomplished using Lagrange multipliers. However, while this results in relatively simple equations when conditioning on moments of distributions, conditioning on information-theoretic quantities results in transcendental equations — making them much harder to solve.

The absence of techniques to efficiently calculate the maximum entropy in these cases is conspicuous; conditioning on the univariate entropies alone is equivalent to assuming the variables are independent, a widely used result, but a generalisation to a wider array of information-theoretic terms has not been forthcoming to the best of our knowledge. In^30^ we introduced a method to address this issue using the set-theoretic formulation of information theory. Here, we extend our maximum entropy technique to continuous random variables and vastly expand the types of information-theoretic quantities one can condition on, compared to the univariate entropies and bivariate mutual informations discussed in ref. 30.

**Figure 1:**
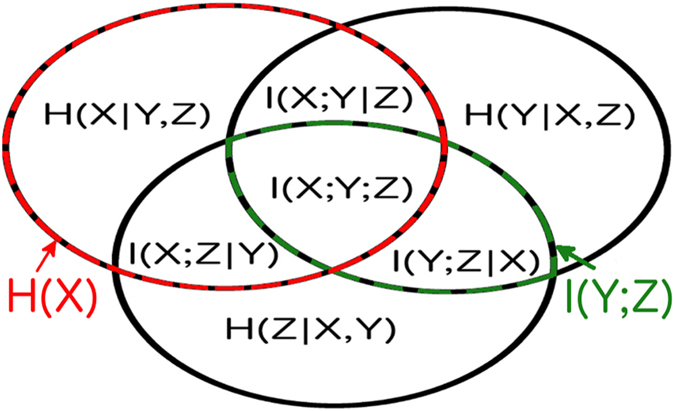
The information diagram for three variables. It contains 7 regions corresponding to the possible combinations of 3 variables, with their corresponding information-theoretic quantities defined in the text. The univariate entropy *H*(*X*) is the sum of all the regions in the red and black striped circle, and the mutual information *I*(*Y*; *Z*) is the sum of all the regions in the black and green striped oval.

The set-theoretic formulation of information theory maps information-theoretic quantities to regions of an information diagram^34^, which is a variation of a Venn diagram. The information diagram for three variables is shown in Fig. 1 with the associated information-theoretic quantities labeled: entropy, $H(X)=-\sum p(x)\mathrm{log}(p(x))$; conditional entropy, $H(X|Y,Z)=-\sum p(x,y,z)\mathrm{log}(p(x|y,z))$; mutual information, $I(X;Y)=\sum p(x,y)\mathrm{log}(p(x,y)/(p(x)p(y))$; conditional mutual information, $I(X;Y|Z)=\sum p(x,y,z)\mathrm{log}(p(x;y|z)/[p(x|z)p(y|z)])$; multivariate mutual information, $I(X;Y;Z)=I(X;Y)-I(X;Y|Z)$. Note that we use the convention $p(x,y,z)=P(X=x,Y=y,Z=z)$.

In general, we consider *N* random variables ${X}_{1},{X}_{2},\ldots ,{X}_{N}$. In the information diagram, any random variable *X*_*i*_ is represented by a set ${\tilde{X}}_{i}$. Notably, it is possible to define a (signed and unique) measure *μ* on the field *F*_*N*_ generated by the sets ${\tilde{X}}_{1},{\tilde{X}}_{2},\ldots ,{\tilde{X}}_{N}$, that maps the measures of sets in the field *F*_*n*_ and the Shannon’s information measures related to the variables *X*_*i*_. In particular, for any (not necessarily disjoint) subsets $G,G^{\prime} ,G^{\prime\prime} \subseteq \mathrm{\{1,2,}\ldots ,N\}$ it holds that:

1 $$\mu ({\tilde{X}}_{G}\cap {\tilde{X}}_{G^{\prime} }-{\tilde{X}}_{G^{\prime\prime} })=I({X}_{G};{X}_{G^{\prime} }|{X}_{G^{\prime\prime} }),$$

which gives as special cases:

$$\mu ({\tilde{X}}_{G}\cap {\tilde{X}}_{G^{\prime} })=I({X}_{G};{X}_{G^{\prime} }),$$

$$\mu ({\tilde{X}}_{G}-{\tilde{X}}_{G^{\prime\prime} })=H({X}_{G}|{X}_{G^{\prime\prime} })$$

and

$$\mu ({\tilde{X}}_{G})=H({X}_{G}\mathrm{).}$$

Due to this equivalence between the Shannon information quantities and the measure *μ*, information-theoretic quantities can be conveniently written as sums of the ‘atoms’ of information diagrams. The *atoms* of the diagram are defined as sets of the form ${\cap }_{i=1}^{N}{Y}_{i}$, where *Y*_*i*_ is either ${\tilde{X}}_{i}$ or ${\tilde{X}}_{i}^{c}$, the complement of ${\tilde{X}}_{i}$. For instance for the three variable case shown in Fig. 1 we can see the decompositions into atoms:

2 $$I(Y;Z)=I(Y;Z|X)+I(X;Y;Z)$$

3 $$H(X)=H(X|Y,Z)+I(X;Y|Z)+I(X;Z|Y)+I(X;Y;Z\mathrm{).}$$

Our approach in tackling the maximum entropy problem is (instead of constructing the maximum entropy distribution explicitly) to construct the information diagram with the largest entropy given the constraints. This intuitively corresponds to creating the maximally disjoint diagram. For example, conditioning on the univariate entropies alone results in the diagram being completely disjoint, i.e., the maximum entropy is the sum of the univariate entropies — a well known result. However, when conditioning on other terms, such as mutual informations, calculating the maximum entropy is no longer straightforward. Nevertheless, given the constraints in the form of Shannon information quantities (that are sums of measures of atoms in the information diagrams), we can search for the maximum entropy using linear optimization procedures. Notably, any set of information-theoretic quantities can be used as constraints with our method — as long as they can be written as a linear function of the atoms of the information diagram. We illustrate this further in the section Estimating Connected Information where we condition on *k*-variate entropies.

In addition to any constraints one chooses, for discrete variables, the measures of atoms of the information diagram must satisfy specific inequalities for the diagram to be valid, i.e. for there to exist a probability distribution with the corresponding Shannon information measures. To obtain a useful maximum entropy estimate, these should be included in the optimization problem definition. An important set of these inequalities are the so-called Shannon inequalities, that can be constructed from elemental inequalities of the following two forms:

4 $$H({X}_{i}|\{{X}_{1},{X}_{2},\ldots {X}_{N}\}\backslash {X}_{i})\ge 0$$

and

5 $$I({X}_{i};{X}_{j}|{X}_{G})\ge \mathrm{0,}$$

where *i* ≠ *j*, and $G\subseteq \mathrm{\{1,2,}\ldots N\}\backslash \{i,j\}$. This is a minimal set of inequalities as no inequality is implied by a combination of the others. Each of these inequalities can also be written as the sum of atoms in their region. This is trivial for inequalities like Eq. (4) since all $H({X}_{i}|\{{X}_{1},{X}_{2},\ldots {X}_{N}\}\backslash {X}_{i})$ corresponds to atoms. There will also be $(\frac{N}{2})$ inequalities like Eq. (5) that correspond to atoms of the diagram. For four variables a nontrivial decomposition into atoms of an Eq. (5) inequality is

6 $$I({X}_{1};{X}_{2}|{X}_{3})=I({X}_{1};{X}_{2}|{X}_{3},{X}_{4})+I({X}_{1};{X}_{2};{X}_{4}|{X}_{3})\ge 0.$$

Less well known, there also exist the so-called non-Shannon inequalities for *N* ≥ 4, which are not deducible from the Shannon inequalities^34^. While it is possible, in principle, to include these in our maximization, they have not yet been fully enumerated. Therefore, we restrict the set of inequalities we use to the Shannon inequalities. As the resulting diagram may thus violate a non-Shannon inequality, there may be no probability distribution that corresponds to the diagram. However, the diagram would still provide an upper bound on the entropy given the constraints.

For a large class of diagrams we do know our bound is achievable. For example, for any diagram where all the atoms are non-negative, it is possible to construct a set of variables that satisfy it (see Theorem 3.11 in ref. 34). It is easy to see from the proof that there will in fact be an infinite number of distributions satisfying the diagram in these cases. There will of course also be many diagrams with negative regions that are also satisfiable.

We have now shown that the task of finding the maximum entropy, conditioned on the information-theoretic quantities discussed here, as well as the elemental Shannon inequalities, can be solved using linear optimization. Each constraint will take the form of a linear equality or inequality, as in Eqs (2 and 6), and we maximize the N-variate entropy by maximizing the sum over all *A* atoms of the information diagram.

Our method is free of distributional assumptions, finding the maximum entropy possible for variables of any cardinality given only information-theoretic constraints. This can result in the maximum entropy diagram being unconstructable for low cardinality variables, even though it is achievable for higher cardinality ones. However this does not seem to be a large issue in practice, as can be seen in our results in ref. 30.

Given information-theoretic constraints of the type we have been discussing, it is just as easy to use linear optimization to find the minimum possible entropy as it is to find the maximum. The minimum entropy diagram is much more likely to have negative atoms though, so our constructive proof of existence is not likely to hold in these cases. Analogous to the maximum entropy diagram, the minimum diagram will still represent a lower bound on the possible entropy. We focus on the maximum case because of its use in statistical physics, and more generally in statistical inference.

## Network Inference

Our maximum entropy estimate allows for the inference of the conditional mutual information between every pair of variables conditioned on all remaining considered variables. These carry the information about the direct connections between the different variables. Conditional mutual informations are typically estimated in a vastly different way^35^ and have also been used to detect causal connections with some success^35^ — though there are fundamental issues in the implementation of reconstructing the underlying time graphs. More importantly, the latter approach to estimate the conditional mutual information becomes increasingly hard as the number of variables and their cardinality go up, due to the exponentially increasing phase space. Our method can help overcome these fundamental issues as well as the sampling issue by not estimating the conditional mutual informations directly, but by finding the values of the conditional mutual information consistent with the measured pairwise mutual informations and univariate entropies when the joint entropy is maximized.

In the process of estimating the maximum entropy, the linear optimization computes all the atoms of the information diagram. This includes the conditional mutual information (CMI) between every pair of variables conditioned on all other variables, e.g., *I*(*X*;*Y*|*Z*) in Fig. 1. This can be interpreted as the level of direct pairwise interaction between components of a dynamical system, and thus be used as a novel method for inferring direct connectivity. In the following section we show how network inference using our entropy maximization, conditioned on mutual informations and univariate entropies, outperforms network inference using mutual informations alone. To benchmark our method’s performance we utilize the Kuramoto model as a paradigmatic dynamical system with non-linear coupling.

### The Kuramoto Model

The paradigmatic Kuramoto model was introduced in ref. 36 and consists of *N* phase oscillators that are coupled in a particular topology. The *i*th oscillator’s phase is given by *θ*_*i*_ and its dynamics are described by

7 $$\frac{\partial {\theta }_{i}}{\partial t}={\omega }_{i}+\frac{K}{N}\sum _{j=1}^{N}{\sigma }_{ij}\,\sin ({\theta }_{j}-{\theta }_{i})+{\eta }_{i}(t\mathrm{).}$$

Here *ω*_*i*_ is the natural frequency of the oscillator, and *η*_*i*_(*t*) a random noise term drawn from a Gaussian distribution with correlation function $\langle {\eta }_{i}(t),{\eta }_{j}(t^{\prime} )\rangle =G{\delta }_{ij}\delta (t-t^{\prime} )$, where *G* determines the amplitude of the noise. *K* represents a uniform coupling strength between interacting oscillators, and *σ*_*ij*_ ∈ {0, 1} represents the coupling matrix of the network, where *σ*_*i*,*j*_ = 1 for connected oscillators. The interactions are always taken to be bidirectional, i.e. *σ*_*ij*_ = *σ*_*ij*_. In the following, we focus on the case when the coupling matrix is chosen as a realization of an Erdös-Rènyi random graph^37^ of link density *ρ* (the number of links divided by the number of possible links), with a fixed number of links. The inference problem is then to reconstruct *σ*_*ij*_ from the measured time series of phases.

The time series are generated using the Euler-Maruyama method with a step size *dt* = 2^−6^ and noise amplitude *G* = 0.05. The data gets resampled such that only every 8th time step is used, and a transient of *T*_*trans*_ = 50 is removed. These parameters are used throughout when discussing the Kuramoto model. Unless otherwise stated the network size is *N* = 12, the integration time *T* = 50,000, the coupling strength *K* = 0.5, and the number of links in the network is 12 (which corresponds to each node having an average of 2 neighbors and a link density of *ρ* ≈ 0.18).

The data is discretized using equiquantal (equiprobable) binning into *n* = 3 states. Numerical tests (using *n* = 5 and *n* = 7) have indicated that larger cardinalities can improve the performance, given that the used time series is long enough. The intrinsic frequencies are drawn from a uniform distribution on the interval [Ω, 3Ω] with ${\rm{\Omega }}=20\cdot \frac{\rho }{N}$. For higher values of *ρ* synchronization effects would be expected at lower coupling strengths. To counteract this, the frequency scale increases with *ρ*. The distribution is shifted away from zero to sample through the phase space more quickly, i.e. avoid oscillators that stay in just one bin throughout the system’s time evolution. For significantly shorter time series (an order of magnitude or greater) the statistics become too poor to reliably estimate the entropies. This however depends on the system being looked at as well as the discretization used, and does not apply universally. In the section Resting-State Human Brain Networks we outline one way to test if there is sufficient data to carry out our analysis.

### Inferring the Network

The basic method to infer a network using our method is to add a link between every two oscillators with a nonzero inferred CMI. However, this results in the inferred link density being relatively independent of the actual link density of the network, as the maximum entropy solution generally provides relatively sparse networks. This is a result of the method generally having at least one zero CMI for all subsets of variables greater than two, so for example this method is unlikely to infer any triangles, see Proofs section at the end. The network inference can be further amended by thresholding either the resulting CMI matrix, or the MI values at its input, with the goal of controlling spurious non-zero values stemming from estimation from finite-size samples.

**Figure 2:**
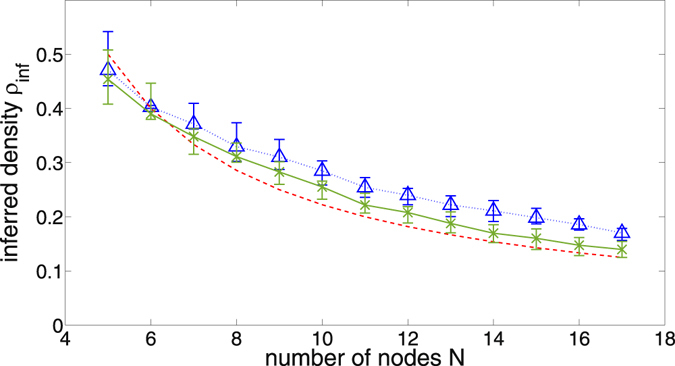
Average inferred link densities *ρ*_*inf*_ as a function of network size, if our maximum entropy method is applied using unthresholded estimated mutual informations. The actual network densities are *ρ* = 0.1 (blue triangles and dotted line), and *ρ* = 1 (green Xs and solid line). The red dashed curve is the density of a network in which every node interacts with two neighbors on average, and given for comparison. The inferred density is calculated as the number of inferred links over the number of possible links, $(\frac{N}{2})$. Each curve is generated from 100 realizations of natural frequencies and coupling matrices with given density *ρ*. The error bars indicate the 25% and 75% quantiles.

Figure 2 shows the inferred link density of the basic method for varying network sizes, and illustrates that there is a maximum achievable inferred link density for a given network size. If a network of higher density is analyzed, the method will fail to identify some links. If in contrast a network is analyzed that is sparser than the average density inferred by the method, our findings necessitate the use of a threshold to reduce the detection of spurious links (since the measured mutual information will not be strictly zero).

To speed up the optimization we use a strictly stronger set of inequalities here, where it is assumed that every atom is non-negative. This provides a lower bound for the maximum entropy. If interactions are truly described by bivariate interactions only, then negative atoms are expected to be negligible, as they would indicate higher-order interactions.

Indeed, for the current Kuramoto model, we have already established that a distribution with strictly pairwise interactions constitutes a good model for the full system^30^. In particular, numerical comparisons at smaller system sizes have indicated that this is indeed a viable approximation. To that end 100 realizations with an integration length of *T* = 10,000 each (the remaining parameters being the same as outlined in the previous subsection The Kuramoto Model) were evaluated at a system size of *N* = 9 using both the exact constraints and the approximate ones. The biggest relative error of the approximate maximum entropy estimate was 0.087%.

**Figure 3:**
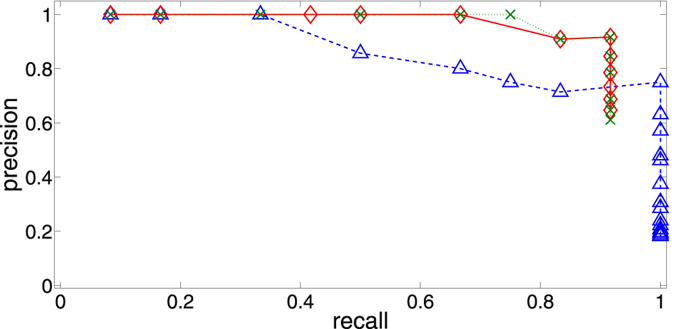
Representative precision/recall curves: Thresholding of MI matrix alone, and not using any maximum entropy method (blue triangles and dashed line); thresholding of CMI matrix obtained using our maximum entropy method on the (unthresholded) mutual informations (green Xs and dotted line); thresholding of mutual informations, and then using the maximum entropy method (red diamonds and solid line).

As mentioned earlier, there are two obvious options for thresholding: either threshold the mutual informations and then apply our maximization procedure, or apply our maximum entropy method first and then threshold the CMI matrix. We have tested both approaches while varying the ‘global’ threshold applied. Our assessment of the method’s performance is based on the precision (ratio of correctly inferred links to all the inferred links) and the recall (ratio of correctly inferred links to the number of links in the real network) (see for example^38^). These measures are particularly well-suited since they are also appropriate for the case of high link density, where our method should detect the ‘backbone’ of the network even though it fails to identify some links correctly. As shown in Fig. 3, using these evaluation metrics neither approach seems to be superior over the other. Both ways generally improve the performance of merely thresholding the mutual information without using any maximum entropy method at all.

**Figure 4:**
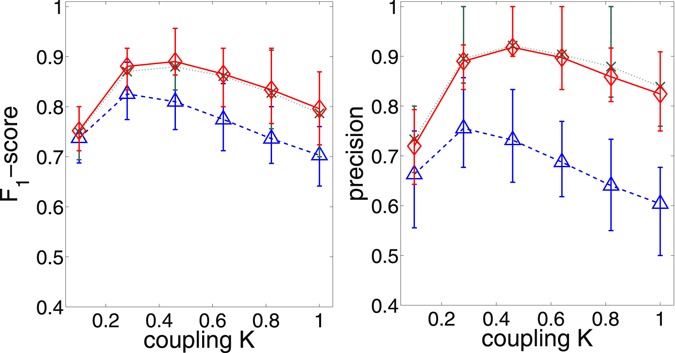
For different thresholding methods the global threshold has been picked that leads to the highest *F*_1_-score (left). The precision corresponding to that threshold is also plotted for comparison (right). The symbols are the same as in Fig. 3. The process has been applied to an ensemble of 100 realizations (with networks and frequencies being randomized for each realization and the same ensemble being studied at each *K*), and the averages are shown. The error bars indicate the 25% and 75% quantiles.

To make this observation more quantitative, it is useful to have a single real number valuation metric to compare performances. We have chosen the *F*_1_-score^38^, defined as ${F}_{1}=2\cdot \frac{precision\cdot recall}{precision+recall}$, because it treats precision and recall symmetrically. From Fig. 4 it is apparent that the best performance is achieved at *K* ≈ 0.5. Considering the coupling is given as $\frac{K}{N}=\frac{0.5}{12}\approx 0.042$ which is of the same order of magnitude as the noise *G* = 0.05, this indicates that our method performs particularly well in the weak coupling regime where no oscillators are synchronized.

Figure 4 also shows that a generally higher precision and *F*_1_-score can be achieved using our method as an additional step after the mutual information thresholding, only partially compromising the recall. The problem of finding a suitable global threshold that actually achieves that performance remains open. Next, we outline a surrogate based method of finding a non-global threshold that displays a performance comparable to the global thresholding discussed above.

A problem for the method’s performance on the Kuramoto model is posed by the fact that two disconnected nodes can have a high estimated mutual information, in a finite time series, if their effective frequencies are close to each other. To account for this we generate surrogates that preserve these effective frequencies as well as the oscillator’s autocorrelations. First the effective frequencies are removed from the time series, subtracting from each oscillator the linear function that interpolates between the initial and final value of their unwrapped phase. That way each oscillator’s time series begins and ends at the same value. In the next step, the Iterative Amplitude Adapted Fourier Transform Algorithm (IAAFT)^39^ is applied to these linearly detrended time series. As a last step, the trends are added back in and for each oscillator a random number uniformly drawn from 0 to 2*π* is added to every value of the time series. This corresponds to randomizing the initial conditions. The mutual informations between the so obtained time series are estimated in the same way as before. This provides an estimate for the mutual information for each pair of oscillators that is not due to their coupling.

To obtain a (local) threshold, a statistical significance level has to be chosen. Since higher significance levels require more surrogate series, the problem can become very computationally expensive. In^11^ the authors suggested the following method for choosing the *p* value: pick *p* so that we expect to keep on average one false link with this threshold. Following this heuristic, we assume that good performance can be expected in the regime of *p* ≈ 98.5%, because there are $(\frac{12}{2})$ = 66 possible links in our system and $\frac{1}{66}\approx \mathrm{1.5 \% }$.

**Figure 5:**
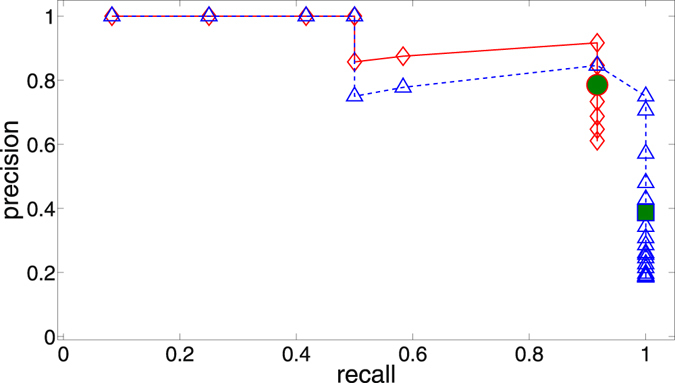
A single typical realization of a precision/recall curve for only thresholding the mutual information (blue triangles and dashed line) and the maximum entropy method being applied to thresholded mutual informations (red diamonds and solid line), similar to Fig. 3. The blue square filled green shows the performance of applying local thresholds to the mutual informations and the red circle filled green the performance of additionally applying our maximum entropy method. The thresholds are determined by the surrogate method discussed in the main text using the *p* = 98.5 percentile. 700 surrogates were generated.

As Fig. 5 indicates, the surrogate-based local thresholding method achieves good performance after our maximum entropy method is applied. This is substantiated by examining an ensemble of such systems as shown in Table 1, clearly establishing the benefit of our maximum entropy method.

**Table 1:** Performance of local thresholding averaged over 100 realizations with *T* = 10,000 and *p* = 90% using 100 surrogates each.

	precision	recall	*F*_1_-score
mutual informations only	0.369	0.997	0.534
with maximum entropy method	0.772	0.834	0.789

The CMI achieved a higher *F*_1_-score in all but 3 cases. The average difference in performance was Δ*F*_1_ = 0.255(+0.079, −0.045) with 25% and 75% quantiles given.

## Estimating Connected Informations

Next we show how our method can be used to nonparametrically estimate connected informations^31^, which are useful for estimating the relevance of higher-order interactions in sets of variables. The connected information of order *k* is,

8 $${I}_{C}^{(k)}(X)=H[{\tilde{P}}^{(k-\mathrm{1)}}(X)]-H[{\tilde{P}}^{(k)}(X)],$$

where ${\tilde{P}}^{(k)}(X)$ is the maximum entropy distribution consistent with all *k*-variate marginal distributions of the N-variate random variable $X=({X}_{1},{X}_{2},\ldots ,{X}_{N})$.

As the number of variables and their cardinality increases, current techniques to estimate these values from probability distributions can suffer from lack of samples due to the exponentially increasing phase space, as well as quickly become computationally intractable. One option to obtain a tractable estimate of the connected information is to compute the quantity:

9 $${I}_{C}^{\langle k\rangle }(X)=H[{\tilde{P}}^{\langle k-1\rangle }(X)]-H[{\tilde{P}}^{\langle k\rangle }(X)],$$

where ${\tilde{P}}^{\langle k\rangle }(X)$ is the maximum entropy distribution consistent with selected moments of the N-variate random variable $X=({X}_{1},{X}_{2},\ldots ,{X}_{N})$. Typically just the first two moments are used, which ensures the cross-correlations will be preserved. As an alternative, we propose the expression

10 $${I}_{C}^{[k]}={\tilde{H}}^{(k-\mathrm{1)}}-{\tilde{H}}^{(k)},$$

where ${\tilde{H}}^{(k)}$ is the maximum entropy consistent with all the one through *k*-variate entropies.

In order to estimate ${\tilde{H}}^{(k)}$ we use our method, putting constraints on the one through *k*-variate entropies and Shannon-type inequalities. This formulation has three advantages: 1) estimating the *k*-variate marginal distributions can be problematic due to insufficient data, whereas much better estimates of the *k*-variate entropies may be available such as^40^, as we show in the section Undersampled Regime; 2) this equation is easily estimated using our maximum entropy estimation, which can offer significant computational speedups over existing techniques, as we show in the section Computation Time; 3) this can be estimated given just the information-theoretic quantities independent of any specific knowledge of the underlying distributions.

Note that we can indeed use our linear optimization procedure here due to the linearity of the concerned information measures in the measures of individual atoms: let *A*_*G*_ be the set of all atoms that lie in the set ${\tilde{X}}_{G}$ corresponding to the joint entropy *H*(*X*_*G*_). The univariate entropy *H*(*X*_*i*_) is the sum of measures of all atoms within the corresponding region: $H({X}_{i})={\sum }_{a\in {A}_{\{i\}}}\mu (a)$. Similarly, the bivariate entropy *H*(*X*_*i*_, *X*_*j*_) is $H({X}_{i},{X}_{j})={\sum }_{a\in {A}_{\{i,j\}}}\mu (a)$. This is easily generalized to the *N*-variate entropy: $H({X}_{G})={\sum }_{a\in {A}_{G}}\mu (a)$. Therefore, we can use any *k*-variate entropy as a constraint in the linear optimization problem.

It is important to realize though that Eqs (8) and (10) differ in that the latter does not constrain the cardinality of the variables and could violate the non-Shannon inequalities as discussed in the section Method. Therefore, it will always be the case that ${\tilde{H}}^{(k-\mathrm{1)}}\ge H[{\tilde{P}}^{(k-\mathrm{1)}}(X)]$. In the examples we have examined^30^, as well as in the illustrative example we give next, this does not seem to appreciably affect the results however.

### Illustrative Example

The quintessential example of an entirely 3-variate interaction is the Exclusive OR (XOR) gate when the inputs are chosen uniformly and independently, the truth table of which is given in Table 2. Any pair of variables taken alone appear to be independent, though given the states of two the state of the third is uniquely determined. This can be generalized to an *N*-variate relationship by taking *N* − 1 independently generated random variables uniformly drawn from the set {0, 1}, and the *N* th their sum modulo two. We can also generalize to arbitrary cardinalities, *C*, by drawing the *N* − 1 variables independently and uniformly from the set {0, .., *C* − 1}, and the *N* th is now their sum modulo *C*.

**Table 2:** Truth table for an Exclusive OR (XOR) gate, where the inputs are *X* and *Y*, and the output is *Z*.

*X*	*Y*	*Z*
0	0	0
1	0	1
0	1	1
1	1	0

We now show that in these cases our nonparametric connected information ${I}_{C}^{[k]}$, will return the same result as the parametric ${I}_{C}^{(k)}$. Given a set of *N* variables with cardinality *C*, and an *N*-variate interaction of the type discussed above, the joint entropy of any set of *k* < *N* variables will be the sum of the univariate entropies, $H({X}_{G})={\sum }_{i\in G}H({X}_{i})$. This means ${I}_{c}^{(k)}={I}_{c}^{[k]}=0$ for *k* < *N*. For *k* = *N*, both ${\tilde{H}}^{(k)}$ and $H[{\tilde{P}}^{(k)}(X)]$ are the true *N*-variate entropies, and ${I}_{c}^{(k)}={I}_{c}^{[k]}=H({X}_{i})$. We can see from this that both methods will also return the same result for a system of *N* variables that is composed of independent sets of *v* variables with *v*-variate relationships, where *v* is allowed to differ between sets, e.g. two XOR gates, where *N* = 6 and *v* = 3 for both sets.

### Resting-State Human Brain Networks

To illustrate the applicability of the described methodology in real-world data situations, we apply it to neuroimaging data, in a similar context as in the recent study by Watanabe *et al*.^32^. In particular, we want to assess to what extent the multivariate activity distribution is determined by purely bivariate dependence patterns. This is of relevance because the use of bivariate dependence matrices, particularly of pairwise correlations, is currently a prominent method of characterizing the brain interaction structure. If pairwise relationships are sufficient to describe the interaction structure of the brain this would tremendously simplify the task of uncovering this structure. If this were not the case, it would mean that higher-order relationships, as discussed in the beginning of this section (Estimating Connected Informations), would need to be analyzed. As the phase space of the problem grows exponentially as we probe ever higher-order interactions, this would result in us rapidly running out of sufficient data to sample these spaces, and measure the corresponding interactions.

The used data consist of time series of functional magnetic resonance imaging signal from 96 healthy volunteers measured using a 3 T Siemens Magnetom Trio scanner in IKEM (Institute for Clinical and Experimental Medicine) in Prague, Czech Republic. Participants were informed about the experimental procedures and provided written informed consent. The study design was approved by the local Ethics Committee of IKEM. Average signals from 10 regions of the well-known default mode network, and 10 regions of the fronto-parietal network were extracted using a commonly used Automatic Anatomical Labelling (AAL) brain atlas^41^. Standard preprocessing and data denoising was carried out using processing steps described in a previous neuroimaging study^42^. The data were temporally concatenated across subjects to provide a sufficient sample of *T* = 36480 timepoints. The data and the code used for our analysis are available upon request. Each variable was further discretized to 2 or 3 levels using equiquantal (equiprobable) binning. For variables of cardinality two, conditioning on the first two moments is equivalent to conditioning on the bivariate distributions, as we prove in the section Proofs. Therefore, the maximum entropy found using our method will be an upper bound on the maximum entropy using the first two moments when the variables are binary.

Entropies were estimated using the estimator in ref. 40. We tested that we could estimate the full joint entropy by estimating it for increasing sample sizes, and checking that the estimate stabilized for the largest available sample sizes. Moving to larger cardinalities was not possible due to insufficient data available to estimate the full joint entropy of the resting-state networks.

To determine the explanatory power of pairwise measurements we compute the connected information of order two divided by the *total correlation* ${I}_{N}={\sum }_{i}H({X}_{i})-H(X)$, where $0\le {I}_{C}^{\mathrm{(2)}}(X)/{I}_{N}\le 1$. If this ratio is zero it means that there is no additional information in the pairwise measurements beyond what can be gained from measurements of the individual variables; If this ratio is one it means that all the information in the joint probability distribution is contained in the pairwise measurements of the variables.

Our analysis of the default mode network resulted in ${I}_{C}^{\langle 2\rangle }/{I}_{N}=0.97$ and 0.85 for the 2-level and 3-level discretizations respectively - i.e. when conditioning on first and second moments, and ${I}_{C}^{\mathrm{[2]}}/{I}_{N}=0.93$ and ${I}_{C}^{\mathrm{[2]}}/{I}_{N}=1.00$, i.e. maximizing entropy given bivariate mutual informations and univariate entropies. Similarly, for the fronto-parietal network, conditioning on the moments resulted in ${I}_{C}^{\langle 2\rangle }/{I}_{N}=0.98$ and 0.85 for the 2-level and 3-level discretizations, and ${I}_{C}^{\mathrm{[2]}}/{I}_{N}=0.89$ and 0.94 using our method. In both cases we can see that conditioning on the first two moments resulted in a substantial decrease in ${I}_{C}^{\langle 2\rangle }/{I}_{N}$ as the discretization was increased, while the results using our method appear more stable to the discretization. The effect of discretization on both these methods is in accord with the results for nonlinear model systems in ref. 30.

Overall, our findings are consistent with the observations reported in ref. 32 for the 2-level and 3-level discretization of the default mode network and the fronto-parietal network, where the authors conditioned on the first two moments only. For 2-level discretization, they found ${I}_{C}^{\langle 2\rangle }/{I}_{N}=0.85$ and 0.96 for the default mode and fronto-parietal networks respectively. For the 3-level discretization, the ratio dropped to ${I}_{C}^{\langle 2\rangle }/{I}_{N}\approx 0.55$ for both networks. The variation between their specific values and ours — especially for the 3-level discretization — is likely a result of the different regions used to represent both networks in combination with statistical variations starting from different data sets to begin with. This is also supported by the findings for a different brain atlas in ref. 30 In conclusion, both their and our findings indicate that multivariate interactions may play a minor role in the structure of fMRI data. However, the presented entropy-conserving approach provides results more consistent across the discretization choices.

### Direct Connectivity in Resting-State Human Brain Networks

**Figure 6:**
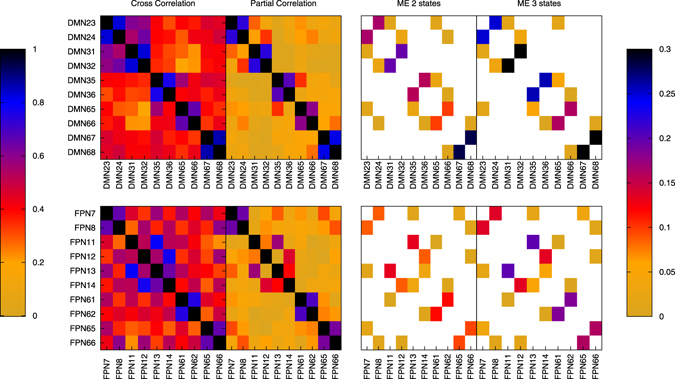
Internal structure of two resting-state brain networks — default mode network (top row) and the fronto-parietal network (bottom row) — represented by their weighted coupling matrix, which has been estimated by different methodologies. The different regions or nodes are labeled with network abbreviation and number according to the AAL brain atlas in ref. 41 The results based on a direct analysis of the original data using cross-correlations as well as partial correlations are shown on the left (note that absolute values are shown), while the results using our maximum entropy approach conditioned on all entropies and mutual informations for the discretized cases of 2 states and 3 states, respectively, are shown on the right. White entries indicate a conditional mutual information of exactly zero.

Taking the bivariate interactions as a first approximation for the full interactions within the default mode network and the fronto-parietal network, we can also infer the direct connectivity as outlined above in the section Network Inference. Since the density of links is expected to be high in both of these networks, we do not apply a threshold; any nonzero inferred CMI constitutes an inferred link. The estimated CMI matrix is shown in Fig. 6 for the cases of two and three states, respectively, alongside the cross-correlation matrix and the partial correlation matrix for the original (non-binned) data, which were estimated using standard MATLAB functions. It can be seen from this that the cross-correlation is sensitive to many indirect relationships that are removed by both our method and the partial correlation.

Most of the entries of the CMI matrices are zero. This is expected, since our maximum entropy methodology generally leads to at least one zero CMI for all subsets of variables greater than two as discussed in more detail in the section Network Inference and the section Proofs. Thus, in the case of a high density of direct connections the CMI matrix should give a representation of the “backbone”, i.e., the most dominant connections of the actual network. As Fig. 6 shows, the backbone of the fronto-parietal network and the default mode network are pretty much robust across the discretization choices — there are only minimal changes related to very weak links.

Moreover, the inferred direct connections and the notion of a “backbone” are consistent with the partial correlation matrix for the original (non-binned) data in the sense that almost all links inferred from the CMI matrix correspond to the strongest partial correlations. By construction, partial correlations allow one to correctly infer the structure of conditional independences in the case of normally distributed data. The observed consistency is hence an indication that in the bivariate approximation the dominating interactions can be considered linear to a large degree. This is in line with the previous finding of relatively marginal deviation from normality in region-averaged fMRI data^42^. Of course, while these marginal deviations from normality of bivariate marginal distributions have been shown not to affect the global graph-theoretical structure of the brain network^43^, there can still exist substantial higher-order interactions not captured by the multivariate normal approximation.

## Computational Advantages

To establish the practical relevance of our maximum entropy method, we now show that it can successfully be applied in the undersampled regime, and that the computation time does not increase with the cardinality of the variables — in fact we show our method can be computed much faster than other techniques for cardinalities greater than 2. These are two issues that severely limit current maximum entropy methods as noted in ref. 44.

### Undersampled Regime

Possibly one of the most exciting applications of our method is in the undersampled regime. It is possible to estimate the entropy of a set of discrete variables with s ~ 2^*H*/2^ samples (where *H* is measured in bits)^40^. This means it is possible to obtain maximum entropy estimates even when the marginal probability distributions have not been sufficiently sampled, as needed to calculate the connected informations in ref. 31.

As an example, consider an Ising type model with probability distribution,

11 $$P({\bf{X}})=\frac{1}{Z}\exp (\sum _{i=1}^{N}{h}_{i}{x}_{i}+\sum _{i > j}{J}_{i,j}{x}_{i}{x}_{j}),$$

where *Z* is a normalization constant. These distributions often arise in the context of establishing the importance of pairwise interactions, because they describe the maximum entropy distribution consistent with the first two moments of a set of variables^3, 30^. Therefore, we would expect the difference between the entropy of the true distribution and the maximum entropy conditioned on the bivariate distributions to be zero.

**Figure 7:**
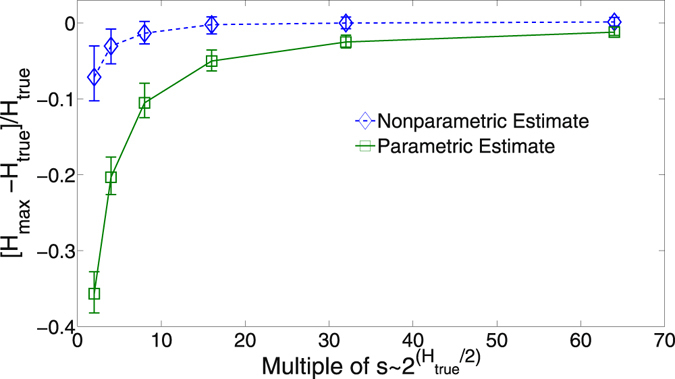
The fractional difference between the maximum entropy estimate and the true entropy using our nonparametric maximum calculated from the univariate and bivariate entropies, as well as estimating the maximum parametrically from the estimated bivariate probability distributions. One hundred distributions of three variables of the form of Eq. (11) were generated with the parameters *h*_*i*_ and *J*_*i*,*j*_ drawn from normal distributions with zero mean and standard deviation 0.1, with each variable having a cardinality of 5. The minimum number of samples needed, *s* ~ 2^*H*/2^, ranged from 4 to 12. The error bars indicate the 25% and 75% quantiles.

At small sample sizes however, the maximum entropy is severely underestimated when conditioning on naively estimated bivariate distributions. On the other hand, a much more accurate estimate of the maximum entropy is obtained when estimating the univariate and bivariate entropies using the estimator in ref. 40, and using these as constraints in our nonparametric method. This is shown in Fig. 7.

### Computation Time

To illustrate the potential computational speedups possible using our methods, we consider Ising type distributions, Eq. (11), again. Specifically, we investigate the dependence on different numbers of random variables, *N*, and variable cardinality. In each case the parameters *h*_*i*_ and *J*_*i*,*j*_ are drawn from a normal distribution with mean zero and variance 0.1.

**Figure 8:**
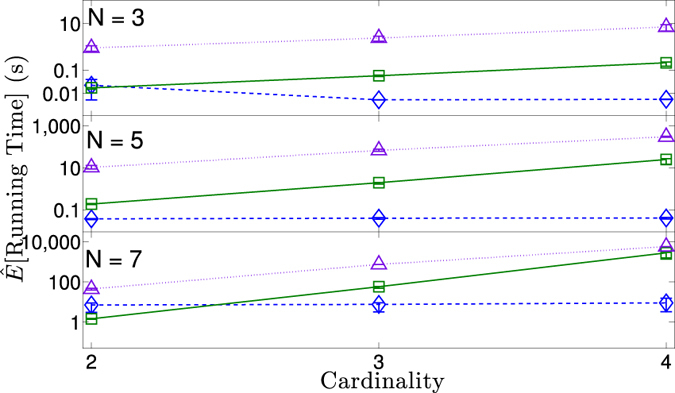
The expected running time for different methods at different variable cardinalities, and number of variables, *N*. The three methods are: our linear optimization method conditioned on mutual informations and univariate entropies (blue diamonds and dashed line); iterative fitting conditioned on the bivariate distributions (green squares and solid line); iterative fitting conditioned on the first two moments (purple triangles and dotted line). For each cardinality and *N*, the distributions and error calculations are the same as for Fig. 7.

Figure 8 compares the runtime of our algorithm with that using iterative proportional fitting^45^, where we show both conditioning on the bivariate distributions and conditioning on the first two moments of the distributions. Since our method only uses information-theoretic quantities as inputs it is not affected by the cardinality of the variables, i.e., if the variables have a cardinality of two or 100 it will have no bearing on how long our method takes to run, as long as the information-theoretic quantities conditioned on are the same. As the other methods do depend on the cardinality of the variables we expect that at ‘some’ cardinality our method will certainly outperform them. In fact, as Fig. 8 shows, only when the variables have a cardinality of two the runtimes are comparable, with our method running orders of magnitude faster at all measured higher cardinalities.

## Discussion and Conclusions

In this paper we extended the method we introduced in ref. 30 to compute the maximum entropy conditioned on a wide range of information-theoretic quantities — beyond the bivariate mutual informations and univariate entropies — using linear optimization. We have also shown how to implement our method with continuous variables, no longer limiting it to discrete ones, making our technique applicable to a much broader range of problems. While there are pathological linear optimization problems whose running time will scale exponentially with the number of variables, *N*, there will always be a slightly perturbed problem such that our method will scale polynomially^46^.

Our method is nonparametric in that it does not generate a corresponding probability distribution. This may result in a diagram for which no probability distribution can be constructed (since it may violate a non-Shannon inequality). However, in the common case where the maximum diagram has only non-negative regions it is necessarily satisfiable.

Using this method we introduced a technique to infer the direct connectivity of a network by estimating conditional mutual informations. We showed that this can be used to improve on the performance of naively thresholding the mutual informations to infer networks of a dynamical system. For the Kuramoto model it was also evident that our method performs particularly well in the relatively weak coupling regime. Note that some other methods have been recently reported to give best performance in a similar Kuramoto model for strong coupling, such as the main method proposed in ref. 13.

Additionally, we demonstrated that our particular thresholding method achieves high precision, while also retaining higher recall than, for example, in ref. 11. There the authors used a method similar in spirit to ours, where they estimated the network based on the thresholded mutual information between all pairs of variables, as well as set the weakest mutual information between every triplet of variables to zero. While they justified this with the data processing inequality, we show in the section Proofs that this also can be justified as a result of maximum entropy estimation, giving further credence to their method. It must be noted however, that bigger system sizes and higher link densities were considered in ref. 11 than can be treated with our presented method.

Indeed, as we noted in the section Inferring the Network, the CMI network corresponding to the maximum entropy distribution is generally sparse. Note that this is actually in line with the Occam’s razor requirement of simple explanation - see the section Proofs for the proof concerning the 3-variable case; showing that the least presumptive solution is for two of three variables to be conditionally mutually independent. The fact that the current method provides network estimates with low clustering, and therefore without small-world properties, is not too detrimental in the light of recent observations that the small-world properties of the functional connectivity of many real-world systems are spurious^47^, as recently documented for the example of brain and climate data^48^.

Motivated by our new ability to easily compute the maximum entropy given information-theoretic constraints, we introduced a nonparametric formulation of connected informations. This can be computed directly using our linearly optimized maximum entropy, and hence has its computational and sampling advantages. For paradigmatic examples of higher-order relationships — which connected informations attempt to detect — we demonstrated that our nonparametric method will give the same result as the standard one.

We have also expanded on our work in ref. 30, where we have now analyzed two resting-state human brain networks built from a different brain atlas. It is highly desirable to know if these networks can be accurately described with pairwise measurements, as it would tremendously simplify their analysis, and is common practice. Previous results indicated that this is the case, but only when the signal is binarized^32^. In both networks analyzed we have shown that conditioning on the first two moments of the distributions exhibits a marked sensitivity to the number of states the system is discretized to. On the other hand our method appears to be more robust to the specific discretization, as was also seen in ref. 30 for the case of the Kuramoto model. This indicates that pairwise measurements can still capture the majority of the complexity of these networks. In this bivariate approximation, the inferred direct connectivity of the backbone of the two resting-state human brain networks using the maximum entropy method is consistent with the results of an analysis of the partial correlations. This indicates not only the robustness of the backbone across significantly different estimators but also that the underlying dynamics can be considered linear to a large degree. This is supported by an analysis of linear surrogates of the fMRI data as well.

Since our method does not require the direct estimate of any probability distribution, we can apply it in the undersampled regime. We demonstrated that in this regime our method offers a much more accurate estimate of the maximum entropy. Additionally, we demonstrated that our method offers computational speedups over competing techniques when the variables have cardinality greater than 2. This makes our techniques perfectly positioned to analyze systems of larger cardinality variables where the size of the phase space can make both computation time and accurate sampling prohibitive.

In conclusion, we have shown that our entropy maximization performs well in the undersampled regime, and for high cardinality variables. This helps resolve two outstanding problems with maximum entropy estimation, as noted in ref. 44. More importantly, we have shown that this method can be applied to real world problems researchers are facing, using network inference and fMRI data as examples. While we have given a few obvious applications for our method, given its broad nature it is our belief that many researchers will find uses for it that we have yet to anticipate.

## Proofs

### Analytical Maximum for *N* = 3

When conditioning on bivariate mutual informations and univariate entropies we have an analytical solution for the maximum entropy when *N* = 3. For three variables we can write the joint entropy as

12 $$H=\sum _{i}H({X}_{i})-\sum _{i > j}I({X}_{i};{X}_{j})+I({X}_{1};{X}_{2};{X}_{3}\mathrm{).}$$

We can see why Eq. (12) is true by imagining the information diagram, and realizing the total entropy must be the sum of all its elements. By adding all the univariate entropies all the conditional entropies in the information diagram are added once, but all the regions of overlap are added multiple times. These multiple counts are then removed when we remove all the mutual informations, but now we remove regions where more then 2 variables overlap too many times. For three variables we then need to add back the triplet region once. It was added three times by the entropies and removed three times by the mutual informations.

Since we are conditioning on the univariate entropies and mutual informations, the only free parameter is

13 $$I({X}_{1};{X}_{2};{X}_{3})=I({X}_{1};{X}_{2})-I({X}_{1};{X}_{2}|{X}_{3}\mathrm{).}$$

This means that the maximum of Eq. (12) will occur when Eq. (13) is maximal. Both *I*(*X*_1_, *X*_2_) and *I*(*X*_1_; *X*_2_|*X*_3_) must be positive, so Eq. (13) can be no greater than the minimum mutual information between *X*_1_, *X*_2_, and *X*_3_.

**Figure 9:**
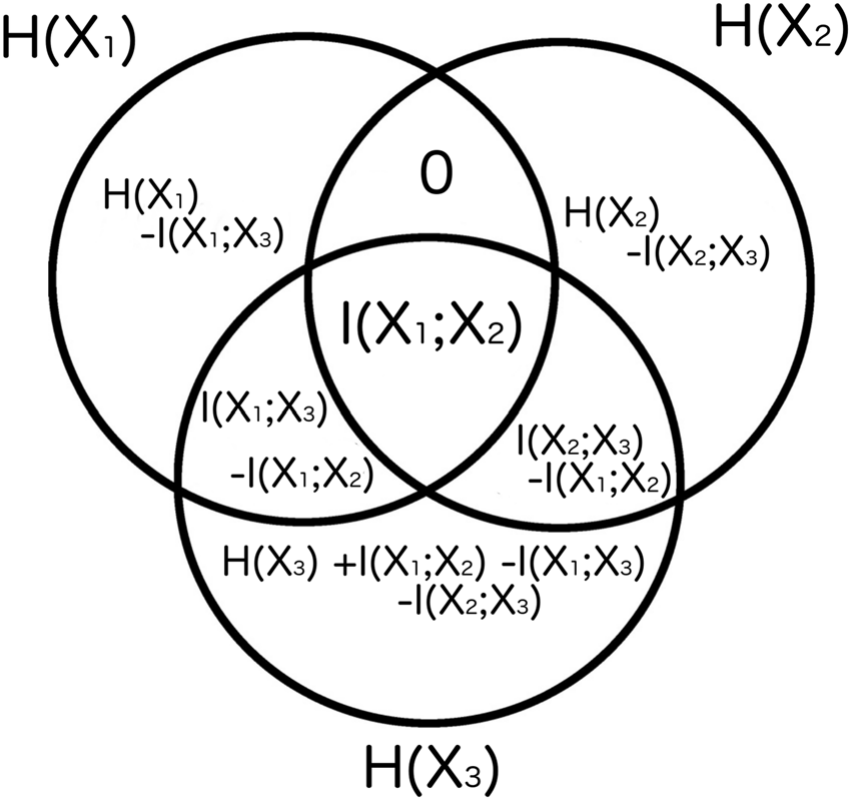
The maximum entropy diagram for three variables if the minimum mutual information between the variables is *I*(*X*_1_; *X*_2_).

We now show that we can always construct this diagram when the variables are discrete since it will only have non-negative regions. Without loss of generality we can define the minimal mutual information to be *I*(*X*_1_; *X*_2_). This results in the information diagram in Fig. 9. By inspection we can see that this diagram satisfies the constraints on the univariate entropies and mutual informations. Since *I*(*X*_1_; *X*_2_) is the minimal mutual information, and all the mutual informations are non-negative, all the regions where multiple variables overlap in the diagram are non-negative. Now we must show that all the conditional entropies in the diagram are non-negative. The mutual information between two discrete variables can not be greater than their univariate entropies, therefore *H*(*X*_1_|*X*_2_, *X*_3_) ≥ 0 and *H*(*X*_2_|*X*_1_, *X*_3_) ≥ 0.

The final part now is to prove that *H*(*X*_3_|*X*_1_, *X*_2_) ≥ 0, which we show is true provided that the constraints are satisfiable. We now look solely at the regions inside *H*(*X*_3_), and look at the affect of adding *ε* to the *I*(*X*_1_; *X*_2_; *X*_3_) region. To conserve the univariate entropy and mutual informations associated with *X*_3_, we must make the following changes

14 $$I({X}_{1};{X}_{3}|{X}_{2})\to I({X}_{1};{X}_{3}|{X}_{2})-\varepsilon $$

15 $$I({X}_{2};{X}_{3}|{X}_{1})\to I({X}_{2};{X}_{3}|{X}_{1})-\varepsilon $$

16 $$H({X}_{3}|{X}_{1},{X}_{2})\to H({X}_{3}|{X}_{1},{X}_{2})+\varepsilon \mathrm{.}$$

We see from this that changing one region in *H*(*X*_3_) necessitates changing all the regions in *H*(*X*_3_). We also see that changing *I*(*X*_1_; *X*_2_; *X*_3_) changes *H*(*X*_3_|*X*_1_, *X*_2_) by the same amount. This means that the largest *H*(*X*_3_|*X*_1_, *X*_2_) can be is when *I*(*X*_1_; *X*_2_; *X*_3_) is also maximal – as in our maximal construction, Fig. 9. Therefore if our constructed case resulted in *H*(*X*_3_|*X*_1_, *X*_2_) < 0 the constraints are unsatisfiable since this is the largest that *H*(*X*_3_|*X*_1_, *X*_2_) can be made.

Figure 9 shows that the maximum entropy, conditioned on bivariate mutual informations and univariate entropies, corresponds to the pair of variables with the smallest mutual information being conditionally independent. This is notable, as it is essentially what is done in ref. 11, where the authors attempt to infer interactions between genes; for every triplet of genes they consider the pair with the smallest mutual information to be independent. While they justify this using the data processing inequality^33^, our proof here lends this procedure further credibility.

### Proof that conditioning on the first two moments is equivalent to conditioning on bivariate distributions for binary variables

Maximizing the joint entropy of a set of binary variables, conditioned on their first two moments, is the same as conditioning on the joint probability distributions. The univariate distributions can be reconstructed from the first moments

17 $$E[X]={x}_{0}p({x}_{0})+{x}_{1}\mathrm{(1}-p({x}_{0}))$$

18 $$p({x}_{0})=\frac{E[X]-{x}_{1}}{{x}_{0}-{x}_{1}}\mathrm{.}$$

This information plus the covariances exactly specify the bivariate distributions. For the bivariate distributions we have

19 $$p({x}_{0},{y}_{0})+p({x}_{1},{y}_{0})=p({y}_{0})$$

20 $$p({x}_{0}|{y}_{0})p({y}_{0})+p({x}_{1}|{y}_{0})p({y}_{0})=p({y}_{0})$$

21 $$p({x}_{0}|{y}_{0})+p({x}_{1}|{y}_{0})=1$$

22 $$p({x}_{0}|{y}_{0})+\frac{p({x}_{1})-p({x}_{1}|{y}_{1})p({y}_{1})}{p({y}_{0})}=1$$

23 $$p({x}_{0}|{y}_{1})+p({x}_{1}|{y}_{1})=1$$

Therefore, for the 2-variable conditional probabilities there is only one degree of freedom when the marginal probabilities are known, which is equivalent to the covariance

$$C[X,Y]={x}_{0}{y}_{0}p({x}_{0},{y}_{0})+{x}_{0}{y}_{1}p({x}_{0},{y}_{1})+{x}_{1}\,{y}_{0}p({x}_{1},{y}_{0})+{x}_{1}\,{y}_{1}\,p({x}_{1},{y}_{1})$$

$$p({x}_{0}|{y}_{0})=\frac{C[X,Y]-{x}_{0}{y}_{1}p({x}_{0})-{x}_{1}\,{y}_{0}p({y}_{0})+{x}_{1}\,{y}_{1}(p({y}_{0})-p({x}_{1}))}{p({y}_{0})({x}_{0}{y}_{0}-{x}_{1}\,{y}_{0}-{x}_{0}{y}_{1}+{x}_{1}{y}_{1})}\mathrm{.}$$

Therefore, maximizing the entropy conditioned on the first two moments of a set of binary variables is equivalent to maximizing the entropy conditioned on their bivariate probability distributions.
